# Gouty Pericementitis

**Published:** 1895-04

**Authors:** Edwin T. Darby

**Affiliations:** Philadelphia


### International Dental Journal.

Vol. XVI. April, 1895. No. 4.

### Original Communications.[1]

### GOUTY PERICEMENTITIS.[2]

BY EDWIN T. DARBY, M.D., D.D.S., PHILADELPHIA.

Mr. President and Fellows of the Academy,—It is often easier to describe a thing than to give it an appropriate name. In the present instance, I am much like the preacher who has prepared his sermon and then fails to find a text to fit it. I am aware that of late the gouty diathesis has been made to stand as godfather and sponsor for nearly all the ills that flesh is heir to. Malaria has been receding into the background and gout and appendicitis are coming to the front. These diseases may not be more prevalent to-day than they were fifty years ago, but science has made such strides that the ability to diagnose them is made easier and their cure more certain. I have not come to you to-night with a new disease and an old name, but rather with an old disease and a new name, and my excuse for doing so is that I cannot find in our present terminology one that seems exactly adapted to the condition which I wish to describe. Gingivitis, phagedenic pericementitis, infectious alveolitis, calcic pericementitis, pyorrhœa alveolaris, and Riggs's disease are all well enough in their way, and some of them are sufficiently correct to be allowed to remain in

---

[1] The editor and publishers are not responsible for the views of authors of papers published in this department, nor for any claim to novelty, or otherwise, that may be made by them. No papers will be received for this department that have appeared in any other journal published in the country.

[2] Read before the Academy of Stomatology.

use. Each of these, as I understand it, is intended to describe a disease which has its beginning at the gingiva, and not near the apex of the roots of teeth. It is not my purpose this evening to enter largely into the physiology, pathology, or even etiology of the diseases under consideration. Another has done this much better, I am sure, than I could do it, and I shall take the liberty of quoting somewhat largely from the admirable paper by Professor C. N. Peirce, and appearing in the INTERNATIONAL DENTAL JOURNAL, April, 1892, and January, 1894. He says, "The pathological state affords the only true basis for a scientific classification of diseases. The term pyorrhœa alveolaris refers only to one symptom, and is, therefore, provisional, and more or less objectionable. As you are aware, Dr. G. V. Black has, with some degree of appropriateness, applied to the disease under consideration the terms gingivitis or phagedenic pericementitis and calcic pericementitis. While these two terms approximate the truth, they do not, to my mind, express the whole truth. From a careful study of the abnormal alveolo-cemental membrane, it appears to me that we must recognize two closely-allied but yet very different pathological states; different, as I shall attempt to demonstrate, in their *etiology*, in their *clinical history*, in their *symptomatology*, and in their *susceptibility to treatment*. . . . In the first place, I believe that while pericementitis is associated with calcic deposit, the origin of the calcic salt and the antecedent condition which determines the locality and character of the deposit, as well as the train of totally distinct symptoms which follow, lead inevitably to the conclusion that the different diseases have thus far been confounded. In one form of pericementitis the origin of the calcic salt is the saliva, and in the other form the blood. The former I shall designate as *ptyalogenic* calcic pericementitis, expressive of the idea that in its origin it is *local*, *peripheral*, and *salivary*. The latter I shall designate as *hæmatogenic* calcic pericementitis, . . . an altogether distinct affection from the preceding, and dependent for its cause upon some morbid material derived from the blood, which will, I think, become apparent from the facts which I hope to adduce." The author from whom I am quoting is to be congratulated upon the nice distinction which he has made and the terms which he has applied to the source from which these deposits arise. Again he says (referring to the hæmatogenic variety of calcic pericementitis), "that in this form of the disease the morbid process begins on the root, and very frequently, if not usually, in the vicinity of the apical extremity, this being in marked contrast to the ptyalogenic form, which always has its origin at the gingival borders." I think it may be said to be generally admitted that two distinct forms of calcic deposits are to be found upon the teeth, the one salivary, the other serumal, or, as Dr. Peirce terms it, hæmatogenic. It is believed by many that the last-mentioned variety is often deposited at or near the apices of the roots of teeth before any lesion or pocket has been formed at the gingivæ. Again, it has been claimed by some that the hæmatogenic variety is an irritant, and is the cause of one form of pyorrhœa alveolaris, and not the sequence,—in other words, that it antedates the inflammation instead of being the result of the inflammatory condition. The alveolo-cemental membrane is a connective tissue, fibrous in character, exceedingly vascular, and resembling the connective tissue found in articulations and joints in other parts of the body. Its function is not unlike that of other connective tissue, and experience has shown that it is susceptible to the same morbific influences. Observation has shown that few teeth are lost from any form of pyorrhœa alveolaris before the thirtieth year, and that the disease cannot be considered common before the fortieth year. Experience and observation has shown that gout and rheumatoid arthritis are not common before the thirtieth year, but that in one form or another they are quite common after the fortieth year. Analysis has shown that the blood of gouty and rheumatic subjects contains a larger percentage of uric acid than is normal, and that in certain conditions of the system this acid, in combination with calcium and sodium, is precipitated and retained in the connective tissue of articulating surfaces, producing all the conditions of inflammation except one,—viz., suppuration. Analysis has shown that the deposit found upon the roots of teeth contain uric acid in combination with calcium and sodium, and that such teeth are frequently found in the mouths of those known to have a gouty tendency, or a pronounced gouty diathesis. But you tell me that these coincidences do not establish truths; that uric acid may exist in larger proportions than is normal in the blood of many persons, and yet there may be no manifestations of gout or rheumatism. Candor compels me to admit the force of the objection, at the same time I can see no reason to suppose that fibrous tissue, wherever located, may not under similar conditions be equally susceptible to the deposition of uric acid. Dr. Alexander Haig, in his work on uric acid in causation of disease, claims that he is able, by certain articles of food, certain beverages and medication, to drive uric acid from the blood to the joints and back again from the joints to the blood; that it is simply a matter of acidity or alkalinity of the blood. Dr. Garrod has shown that the cartilages and fibrous tissues of joints are less vascular and less alkaline than the other tissues or the blood, and since that be so, the arthritis produced by uric acid is a simple matter of solubility, and can be produced at pleasure to almost any extent. But it is not my purpose to enter into this phase of the subject. The object of this paper is to describe a few peculiar cases which have come under my observation during the past fifteen or more years; cases which at the time puzzled me not a little, and even now would bear a little illumination. These cases may not be exceptional, and possibly all of you have seen similar ones. I shall describe them as concisely as possible and trust that you may grasp the salient points of each. Mrs. A., perhaps forty years of age, called, suffering much discomfort from a first superior molar. The tooth was sound with the exception of a minute filling in the masticating surface; all other teeth present in the mouth and no indication of disease elsewhere. The first thing to attract my attention was a tumefaction at or near the apex of the buccal roots. It was quite large and presented much the appearance of an apical abscess just ready for the lance, except that its color was dark red, almost purple in hue. The tooth was somewhat elongated and sore to percussion or pressure. Without a moment's hesitation I decided that the tooth was a devitalized one and, with a spear-pointed drill in the engine, proceeded to make an opening into the pulp-chamber, the gold filling being my starting point. As I approached the pulp my patient gave indication of increasing pain, but supposing that to be caused by my pressure upon an inflamed pericementum, I lessened the pressure and revolved my drill with greater rapidity. You can imagine my surprise and chagrin when I found that I had plunged my instrument into a vital pulp. I said to myself, an anomalous case, indeed, an abscess upon a vital tooth. I then began to look for a cause, but to my surprise there was no break at the gingivæ. No salivary or serumal deposits to be seen, and no pus in what I had taken to be a sac. Baffled and confounded, I applied the ordinary remedies for pericementitis, devitalized the pulp and extirpated it, filled the canals, pronounced myself a careless dentist, and awaited results. Subsequent attacks of a similar character, extending over a period of perhaps five years, rendered the tooth a source of annoyance and discomfort. My patient finally concluded that an empty house was preferable to a bad tenant and it was removed,—the first break in an arch which contained sixteen beautiful teeth. Near the apex of the buccal roots of the tooth in question a deposit of considerable serumal calculus was found, and in my opinion this was the cause of the first attack of pericementitis. I cannot say of my own knowledge that this lady ever had an attack of gout, but I do remember that prior to her death her hands were much disfigured by nodosities in the joints of the fingers. If this lady was a victim of uricacidæmia, it may have taken the form of rheumatic arthritis instead of gout, as generally understood. Garrod says, "It is by no means rare to hear of inflammation of a joint by one practitioner called gout, by another rheumatism, and by a third rheumatic gout." The following case is more satisfactory because the patient is living and I have been able to follow it until the present time.

Miss B., aged about forty-five, called with the second inferior molar somewhat elongated and exceedingly sore to the touch. I observed the same tumefied condition upon the gum near the apex of the root, the same angry red appearance of the mucous membrane. The tooth was a sound one, never having been carious. Remembering my former case I did not attempt to open the tooth, but gave it such treatment as is general in pericementitis. I naturally looked for some exciting cause in the form of calculus, but failed to find any, either upon the tooth in question or other teeth in the mouth. I am positive that there was no pocket at the gingivæ. This attack lasted for several days and gradually subsided, but was followed a year or more later by another, at which time a pocket was apparent and pus exuding from about the neck of the tooth. The tooth was finally lost and serumal deposits found near the apex. Two other molars and a bicuspid in this mouth have had like histories during a period of ten years. Another molar is apparently to be lost in the same manner, for during the past few months I have seen the case to give it such treatment as I could to relieve existing pain. I may say just here that but one tooth in the mouth has been affected at the same time, and there is no appearance of pyorrhœa alveolaris in any part of the mouth.

I have recently learned the following facts, which it seems to me have an interesting bearing upon this case: The father, whom I knew well, had been a lifetime sufferer from gout,—the real old-fashioned kind,—which began with painful manifestations in the big toe and did not often get above the ankle-joints. I remember that years ago he told me that he used Blair's gout pills, made in England, and that he obtained greater relief from these than anything he had ever tried. He had been a patient of mine for twenty years, and has lost many of his teeth from pyorrhœa alveolaris. The daughter, whose case I am describing, has never been in robust health, and has frequently had painful attacks of gout of the stomach. It seems to me that to an unprejudiced mind the proof of an inherited gouty diathesis is almost positive, and, if uric acid has any part in the production of calcic pericementitis, here is a case to warrant the supposition that it was the exciting cause in the attacks narrated.

The case which I am about to describe next would seem to furnish more positive evidences of the dual existence of uræmia, in the form of gout and inflammation of the alveolo-cemental membrane, than either of those previously mentioned. It is that of a bachelor, of about fifty years of age, who called upon me about three years ago, with much the same conditions which I have described in the preceding cases. At that time a bicuspid of the superior jaw was the seat of pain. The gum presented the same swollen and angry red appearance. He complained of great sensitiveness to heat and cold and to pressure. As the tooth was without fillings or cavities of decay, I did not open it, but gave the ordinary local treatment common in cases of incipient pyorrhœa alveolaris (?). It was some days before relief was obtained. At that time there were no other teeth similarly affected. As I remember the case, there was no discharge of pus following the attack. A year or possibly eighteen months subsequently the gentleman called, suffering pain in the same tooth. This was followed by suppuration, and of course a pocket at the gingivæ. Since then two or more teeth have had like histories. Anxious to know whether this individual had the uric acid vice, I inquired of an intimate friend of his and learned the following particulars. He has been a "high liver" for many years; indulged freely in wine, and especially champagnes; is a great sufferer from gout and dyspepsia.

I have reserved until the last a case which impresses me as being of peculiar significance. A gentleman, between sixty and sixty-five years of age, has called upon me several times during the past six months, complaining of great discomfort in a superior molar. It has been somewhat elongated and painful in mastication. There have been no marked indications of gingival irritation, no deposits of calculus, but redness and tumefaction along the buccal aspect of the gum. It had failed to respond to ordinary treatment, and, as other teeth had been lost from similar attacks, the gentleman insisted upon having this one removed. I found deposits of serumal calculus on both buccal roots. In reply to my inquiry as to the presence of gout or rheumatism in the system, he pointed to his feet, which were encased in shoes made of soft kid, and greatly out of proportion to the man's stature. He then said, "I have been a great sufferer from gout for many years. I inherited it from my ancestors. I have thrice been to German spas for relief, and spent the whole of last summer in Carlsbad, hoping to find a cure, and am, indeed, better than in the spring." In reply to my inquiry as to whether he had taken salicylate of soda, he said that he had taken pounds of it, until it had so upset his digestion that he was obliged to discontinue its use. He had also taken lithia in various forms, but with indifferent results. Colchicum afforded the surest relief in exacerbations of the disease, but this was open to the same objection as the salicylate of soda.

I could mention other cases which have had similar histories, but no additional light need be shed upon the subject under consideration. I trust I have not wearied you with these details. Your experience and observation may not have been unlike my own. I have never been satisfied with the theory that all cases of pyorrhœa alveolaris were of local origin, and, while I cannot furnish proof positive that uric acid plays an important part in the formation of the calcic deposits to be found high up on the roots of teeth, I can see no objection to such a theory,—and why, I ask, may it not be so? Why may not the connective tissue forming the alveolo-cemental membrane be the seat of uric acid formations just as often as the joint of the big toe, the ankle, the knee, or the phalanges? The physiology and pathology of such a process is as simple as the pin-point nodules which are left upon the mitral valves of the heart in rheumatism or the concretions of the kidneys. "Inasmuch as all portions of the body have been shown by pathologists to be liable to uric acid deposits, it is not at all strange that the alveolo-cemental membrane, composed largely of connective tissue, should also become a depot for uric acid deposits. It is more than probable that as a predisposing cause there might coexist some impairment in the nutrition of this membrane dependent upon either local mechanical force or some obscure faulty innervation. However this may be, the mere presence of these salts leads to the conclusion that here as elsewhere they are derived from the blood by or through the medium of the lymph-stream. With the absorption of the excess of lymph, the residual salts become precipitated upon the cemental surface." (Dr. Peirce, INTERNATIONAL DENTAL JOURNAL, January, 1894.)

There has been one peculiar manifestation in the cases which have come under my observation, which at first I found it difficult to explain, and that is the absence of suppuration in the first or even the second attacks of pericementitis. There have been heat, redness, swelling, pain, but not always pus. But as I have thought more upon the subject, I have concluded that the formation had not yet reached sufficient quantity to produce tissue-disorganization, and that, like other forms of pericementitis, yielded to bloodletting and counter-irritation.

Since beginning the preparation of this paper, my attention has been directed to an essay which was read by Dr. W. J. Reese, of Galveston, Texas, before the Louisiana State Dental Society, March, 1886, entitled "Uræmia and its Effects upon the Teeth." He begins his paper by the statement that as early as 1880 he became convinced that uric acid was the cause of "grave troubles in the mouth," and gives his reasons for believing that it is one cause of what he terms "phagedæna pericementi." Among other things, he says, "The term pyorrhœa alveolaris is a misnomer. One peculiarity of uric acid is that, while it will produce violent inflammation and intense pain, it rarely causes suppuration, except when in contact with the fluids of the mouth, and not always under these circumstances. Where the saliva does not come in contact, and where it is protected from the air, we have no suppuration on the roots of the teeth." In this particular, Dr. Reese's observation has been similar to my own.

In closing this imperfect paper, I would beg you to bear in mind that it is not intended as a scientific contribution to the etiology of a disease, but rather a description of a few cases which it seems to me cannot be accounted for except upon the theory of a constitutional vice.

